# Quantitative Study of Abdominal Blood Flow Patterns in Patients with Aortic Dissection by 4-Dimensional Flow MRI

**DOI:** 10.1038/s41598-018-27249-9

**Published:** 2018-06-14

**Authors:** Dongting Liu, Zhanming Fan, Yu Li, Nan Zhang, Zhonghua Sun, Jing An, Aurélien F. Stalder, Andreas Greiser, Jiayi Liu

**Affiliations:** 1 Department of Radiology, Beijing Anzhen Hospital, Capital Medical University, Beijing, 100029 China; 2 Department of Medical Radiation Sciences, Curtin University, Perth, 6102 Australia; 3 Siemens Shenzhen Magnetic Resonance Ltd, Beijing, China; 4 MR Collaborations NE Asia, Siemens Healthcare, Shanghai, China; 5 Siemens Healthcare, Erlangen, Germany

## Abstract

The purpose of this study is to evaluate the hemodynamic characteristics of the true lumen (TL) and the false lumen (FL) in 16 patients with aortic dissection (AD) using 4D flow magnetic resonance imaging (MRI) and thoracic and abdominal computed tomography (CT) angiography. The quantitative parameters that were measured in the TL and FL included velocity and flow. The mean area and regurgitant fraction of the TL were significantly lesser at all four levels (p < 0.05); the average through-plane velocity, peak velocity magnitude, average net flow, peak flow, and net forward volume in the TL were considerably higher (p < 0.05). The intimal entry’s size was negatively correlated with the blood flow velocity and flow rate in the TL (p < 0.05) and positively correlated with the average through-plane velocity, average net flow, and peak flow in the FL (p < 0.05); the blood flow indices in the TL were enhanced with an increase in the intimal entry numbers (p < 0.05) and the peak flow in the FL was lowered (p = 0.025); if FL thrombosis existed, the average through-plane velocity and peak velocity magnitude in the TL were substantially higher (p < 0.05). 4D flow MRI facilitates qualitative and quantitative analysis of the alterations in the abdominal aortic blood flow patterns.

## Introduction

Aortic dissection (AD) is a vascular disease resulting from an injury in the aortic wall, and it leads to the creation of a true lumen (TL) and a false lumen (FL) separated by an intimal flap with multiple communicating tears between the lumina^1,2^. The condition is life-threatening, and the patients’ long-term prognosis is poor as the five-year mortality rate is 50%^3^. Morphological and hemodynamic changes are the prime factors that affect the stability and development of AD, and the aortic diameter functions as an important indicator of disease progression^4,5^. As the diameter of the aortic lumen increases, the shear stress is augmented and hence, the risk of aortic dissection and aneurysm rupture escalate. Hemodynamic instability and vascular compromise are established risk factors for mortality^6^. Common risk factors, conditions, and stratified outcomes associated with AD have been published in the International Registry of Acute Aortic Dissection (IRAD)^7,8^.

In recent years, noninvasive imaging techniques, such as multidetector computed tomography (MDCT) and magnetic resonance imaging (MRI) have largely replaced conventional angiography for the diagnosis of AD^9,10^. MDCT possesses a high spatial resolution, thereby facilitating an excellent anatomic visualization of AD; however, it is a modality that relies on ionizing radiation and fails to provide hemodynamic information pertaining to the TL and FL. MRI holds many advantages over CT for studying the morphology and hemodynamics of cardiovascular diseases. Two-dimensional phase contrast MRI (2D PC-MRI) is capable of revealing the flow pattern differences in the true and false lumina^11–13^. Data from previous investigations suggest that variations in flow patterns and hemodynamics may play an important role in predicting the complications and determining the outcomes of AD. However, the drawback is that 2D PC-MRI is limited to imaging a single 2D plane. Recently, improved Cartesian^14^ and radial^15^ four-dimensional (4D) flow-sensitive velocity mapping techniques (4D flow MRI) have been introduced for the evaluation of flow patterns. The purpose of this study is to qualitatively and quantitatively assess the abdominal blood flow pattern in patients with AD using 4D flow MRI. We hypothesize that this technique can serve as a useful noninvasive hemodynamic monitoring method for the patients.

## Materials and Methods

### Patient Population

The Human Research Ethics Committee of Beijing Anzhen Hospital approved this prospective study upon confirming that all the procedures were performed in accordance with the relevant guidelines and regulations, and that informed consent was obtained from all the participants and/or their legal guardians. From January 2016 to March 2017, 16 patients (1 female and 15 males) with sub-acute aortic dissection were recruited. The mean time interval from the diagnosis of the condition to the execution of the 4D flow MRI was 7.6 days (range: 0–14 days). Among the participants, eight had undergone open chest surgery and an equal number had endured interventional procedures. The members were neither allergic to the contrast medium nor had a history of cardiac, hepatic, or renal function impairments. Patient characteristics are summarized in Table 1. All the 16 patients underwent thoracic and abdominal CT angiography (CTA) and abdominal aorta 4D flow MRI imaging in the week preceding the surgery.

**Table 1:** Characteristics of the study population [$\bar{x}$ ± s, n(%)].

Variables		Value
Gender	Male	15* (93.8)
	Female	1 (6.3)
Age		^#^51.19 ± 9.21
AD type	Stanford A	1 (6.3)
	Stanford B	15 (93.8)
Number of entries	1	9 (56.3)
	2	4 (25.0)
	3	3 (18.8)
Thrombus	No	11 (68.8)
	Yes	5 (31.3)
Size of entries (mm)		10.65 ± 5.22
Origin RRA	True lumen	12 (75.0)
	False lumen	3 (18.8)
	Overriding	1 (6.3)
Origin LRA	True lumen	8 (50.0)
	False lumen	5 (31.3)
	Overriding	3 (18.8)
Hypertension	No	2 (12.5)
	Yes	14 (87.5)
CAD	No	15 (93.8)
	Yes	1 (6.3)
Diabetes	No	16 (100)
	Yes	0 (0)
Method of operation	interventional procedures	8 (50.0)
	open chest surgery	8 (50.0)

*N indicates the number of patients; the data in the parentheses are percentages.

^#^Represents mean ± standard deviation.

Number of entries: 1–one intimal tear; 2–one entry tear and one secondary tear; 3–one entry tear and two secondary tears.

Abbreviation: AD–aorta dissection, LRA–left renal artery, RRA–right renal artery

### CTA scanning protocol

The scan was conducted on a 320-row CT scanner (Aquilion One, Toshiba Medical Systems, Ottawara, Japan). Prior to the imaging, 2 ml/kg body weight of contrast medium (Iopamiron, 370 mg/ml; Bracco S.P.A., Italy) was injected through a 20-gauge needle into the antecubital vein at a flow rate of 4 ml/s. Subsequently, 40 ml of saline solution was injected at a flow rate of 4 ml/s. A CT power injector (Ulrich Medical, Germany) was used for both the purposes. Image post-processing and analysis were performed using a Vitrea workstation (Vitrea fx ves 6.0). To minimize the radiation dose, the following CT protocol was implemented: tube voltage = 120 kV, tube current = 400 mA, pitch factor = 0.8 with a rotation speed = 0.3–0.5 s, matrix size = 512 × 512, and reconstructed slice thickness = 1 mm. The scan range was from the entrance of the chest to the iliac artery bifurcation level, and it was completed in one breath hold. Volumetric CT scans were initiated by a triggering threshold of 180 HU in the level of pulmonary artery bifurcation.

The volumetric CT dose index (CTDI_vol_) and dose-length product (DLP) of each examination was archived by the system upon completion of the scan. The effective dose (ED) was calculated using a k-factor of 0.015 mSv/mGy.cm^16^.

### 4D-Flow MRI Acquisition Techniques

4D-flow MRI was performed with a 3 T MR system (MAGNETOM Verio, Siemens Healthcare, Erlangen, Germany) consisting of a 32-channel body matrix coil. The prototype 4D-flow sequence covered the abdominal aorta and utilized prospectively ECG-gated and navigator-triggering techniques. The process parameters were as follows: temporal resolution 47.2 ms, TE/TR = 2.79/5.90 ms, FA = 8°, FOV = (255 × 340) mm^2^, matrix = 115 × 192, slice thickness = 1.80 mm, BW = 491 Hz/px, GRAPPA acceleration factor = 2, approximate imaging time = 6–9 min. Velocity encoding was selected based on 2D phase contrast cine MRI (VENC scout) images obtained in the absence of velocity aliasing, and it was carried out prior to the 4D Flow MRI with the ECG gated gradient echo sequence. The velocity encoding was 90–150 cm/s, and it was determined on the basis of the maximum flow velocity measured in the true lumen.

### Post-processing of CTA and 4D-Flow Data

Flow post-processing and measurements were accomplished by two radiologists with 10 and 22 years of experience in interpreting CT and MR images, and consensus was reached in case of any discrepancy.

**Figure 1:**
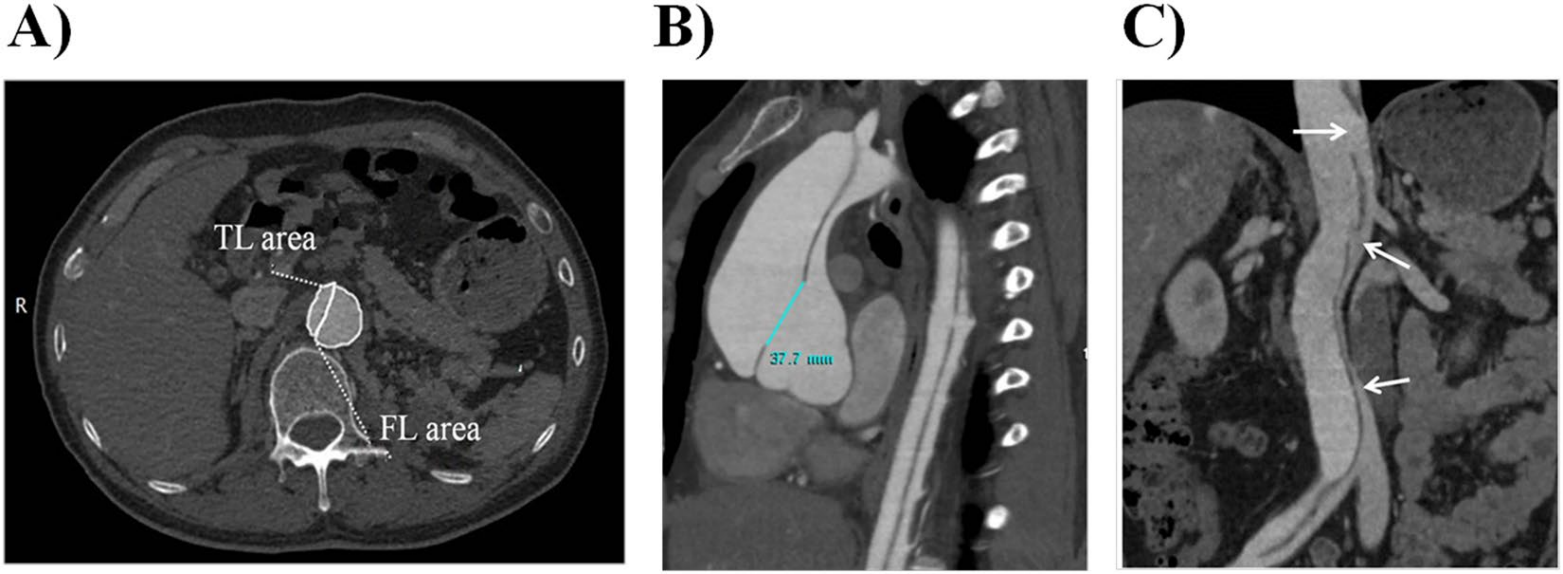
Example of area and size measurement; number of re-entry tears. (**A**) Area measurement of TL and FL. TL = true lumen, FL = false lumen. (**B**) Size measurement of the entry tears. The primary entry tear was located in the ascending aorta. (**C**) MPR image displaying three re-entry tears in the abdominal aorta.

For all the patients, CTA examinations were done in the thoracic and abdominal aorta, as well as in the associated great vessels. A series of 2D and 3D reconstructions including multi-planner reconstruction (MPR), maximum-intensity projection (MIP), and volume rendering (VR) images were generated. The signs associated with AD were identified on the images and these included the mean area of the TL and FL (Fig. 1A), AD types, origin of renal arteries from the AD, FL thrombosis, as well as the size, number, and position of the entry tears measured on MPR images (Fig. 1B,C). From the different MPR images, the maximum diameter was determined based on the size of the entry and re-entry tears in the abdominal aorta.

**Figure 2:**
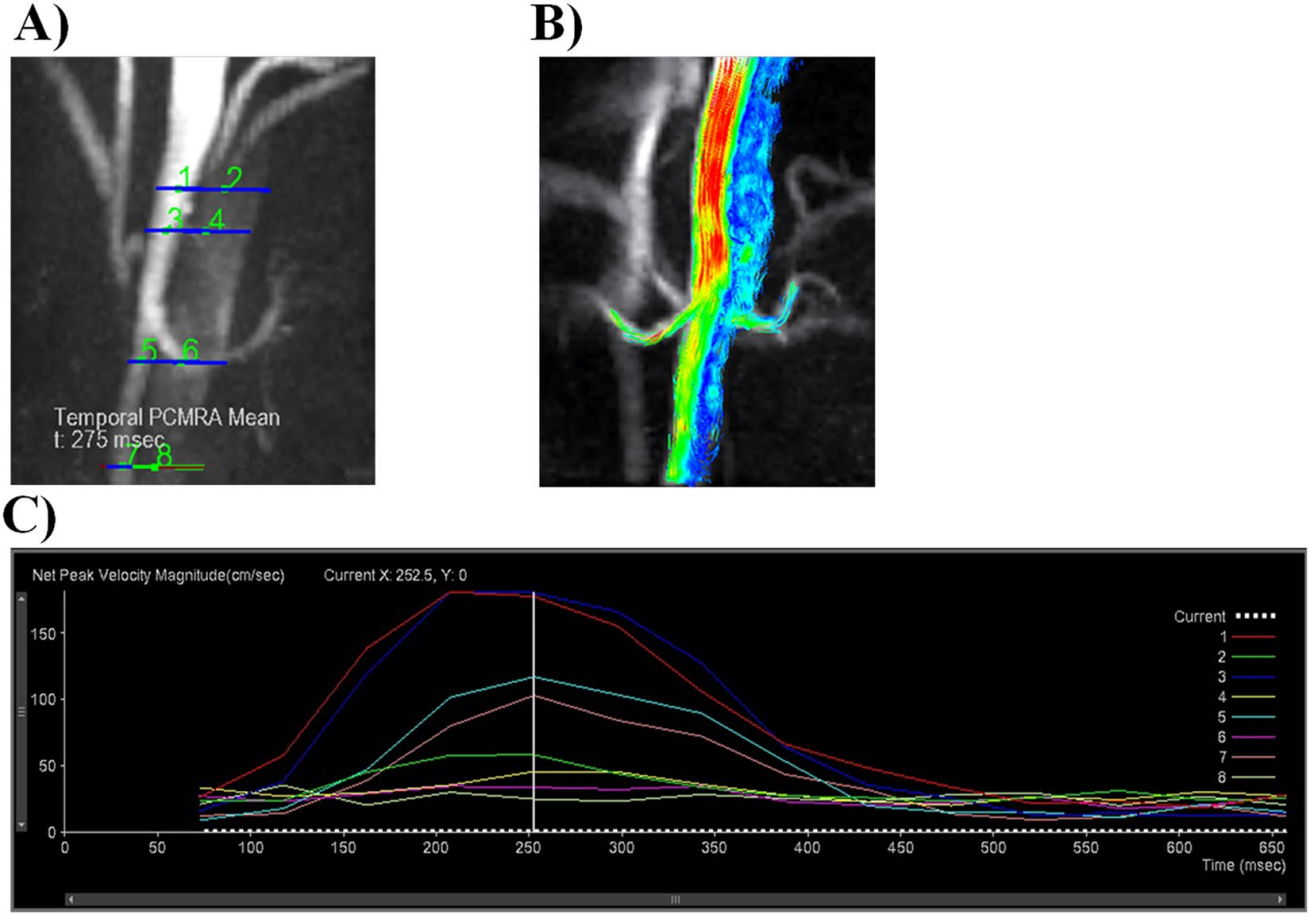
Different levels of flow, velocity, and volume measurements. (**A**) Example of analysis plane selection. TL: 1,3,5, and 7 and FL: 2,4,6, and 8. (**B**) Color-coded streamlined visualization of the faster flow through the TL relative to the FL. (**C**) Time curves of peak velocity magnitude indicating the changes in the blood flow parameters during the cardiac systolic and diastolic phases. They represent the velocity-time curves at different locations in the TL and FL.

The 4D-flow data sets were transferred to a prototype of 4D-flow post-processing software (V2.4, Siemens Healthcare, Erlangen, Germany) for analysis. Four analysis planes transecting the aortic lumen orthogonal to the anticipated main flow direction were manually placed in the following regions (Fig. 2A): (1) celiac trunk, (2) superior mesenteric artery, (3) lower renal artery orifice, and (4) aorta-iliac bifurcation level. The outlines of the TL and FL were defined manually in each axial plane. Odd numbers were assigned to the TL and even numbers to the FL (Fig. 2A). Later, time-resolved images of the 3D velocity vector fields were generated to display the blood flow within the abdominal aorta. Next, 3D streamlines (Fig. 2B) and time-density curves (TDC) (Fig. 2C) of the blood flow parameters were obtained from the 4D datasets. The quantitative parameters that were measured consisted of average through-plane velocity, peak velocity magnitude, average net flow, peak flow, net forward volume, and regurgitant fractions in the TL and FL of each plane.

### Statistical Analysis

The data were entered into the Statistical Package for the Social Sciences (SPSS) software (SPSS, V 20.0 A IBM Corporation, Armonk, NY, USA) for analysis. The continuous variables were presented as mean with standard deviation. The variations in the mean area, average through-plane velocity, peak velocity magnitude, average net flow, peak flow, net forward volume, and regurgitant fractions in the TL and FL were tested by using the paired *t*-test. Pearson correlation analysis and multiple linear regression were applied to analyze the relationship between the blood flow parameters and the various CT imaging variables associated with AD. A p-value of less than 0.05 was considered as a significant difference.

### Informed consent statement

The authors confirm that all the methods were performed in accordance with the relevant guidelines and regulations, and informed consent was obtained from all the participants and/or their legal guardians.

## Results

### General Information and Morphological Characteristics of AD

Both CT and MRI examinations were successfully completed for all the 16 patients and the procedures were devoid of complications. The mean ED was estimated to be 18.03 ± 0.59 mSv (range: 15.69–23.72 mSv). Type A AD was diagnosed in one patient with the intimal tear being present in the ascending aorta, while type B was noticed in the rest of the patients with the tear being located at the distal end of the left subclavian artery or in the descending aorta. Nine patients had one entry, whereas the remaining seven had multiple (2 or 3) entries. All patients had a history of acute chest pain, and the duration from the onset of pain to the preoperative examination was 0.5–15 days. Among these 16 patients, 8 received interventional procedures and the remaining underwent thoracic surgery. Fourteen of the participants had a history of hypertension and one had coronary heart disease. However, none of them had either kidney disease or diabetes (Table 1).

### Qualitative and Quantitative Analysis in the TL and FL

Comparison of the average lumen area and 4D-flow analysis of the TL and FL

**Figure 3:**
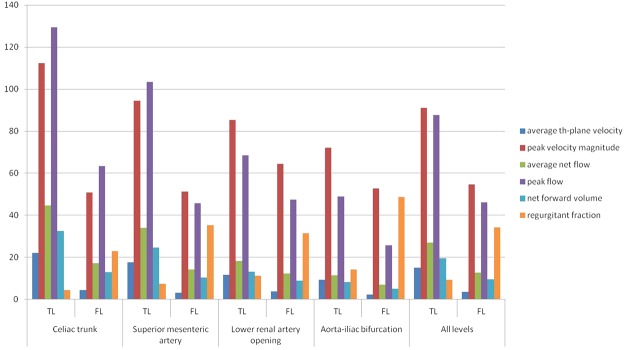
Blood flow parameter variations in different analytical planes of the abdominal aorta.

In all the patients, the average TL area of the four levels was smaller than that of the FL (Table 2). The average through-plane velocity, peak velocity magnitude, average net flow, and net forward volume were significantly higher for the TL in the levels of the celiac trunk and the superior mesenteric artery (P < 0.05). The regurgitant fraction was lower in the TL than in the FL (P < 0.05). However, there was no significant difference between the two regions in terms of the peak flow (P > 0.05). The average through-plane velocity was substantially elevated in the TL at the level of the lower renal artery opening (P < 0.05). The regurgitant fraction was lower in the TL (P < 0.05). There was no significant differences between the TL and FL in terms of the peak velocity magnitude, average net flow, peak flow, and net forward volume (P > 0.05). The average through-plane velocity and peak velocity magnitude were considerably greater in the TL at the aorta-iliac bifurcation level (P < 0.05). The regurgitant fraction was lower in the TL (P < 0.05), but there were no significant differences between the TL and FL in the average net flow, peak flow, and net forward volume (P > 0.05). These 4D flow parameters at different levels are presented in Table 3 and Fig. 3.

**Table 2:** Comparison of the area in the TL and FL.

Level	Area (Mean ± SD, mm^2^)		t	P
	True lumen	False lumen		
Celiac trunk	219.88 ± 113.64	541.39 ± 169.02	−5.95	<0.0001
Superior mesenteric artery	229.24 ± 110.84	485.35 ± 166.23	−4.34	0.0008
Lower renal artery opening	161.14 ± 68.15	357.05 ± 166.28	−3.90	0.0018
Aorta-iliac bifurcation	160.67 ± 100.90	331.01 ± 175.69	−4.02	0.0017
All levels	192.73 ± 102.81	432.46 ± 186.82	−9.08	<0.0001

Data are presented in mean ± standard deviation (SD).

**Table 3:** Comparison of 4D-flow analysis in the TL and FL.

Different planes	average th-plane velocity (cm/sec)		t	P	peak velocity magnitude (cm/sec)		t	P	average net flow (ml/sec)		t	P
	TL	FL			TL	FL			TL	FL		
Celiac trunk	22.05 ± 13.02	4.46 ± 3.17	5	0.0002^▲^	112.37 ± 55.84	50.81 ± 11.82	4.98	0.0002^▲^	44.74 ± 25.8	17.2 ± 14.46	2.63	0.02^▲^
Superior mesenteric artery	17.61 ± 10.58	3.19 ± 2.37	4.52	0.0006^▲^	94.53 ± 48	51.28 ± 17.52	4.01	0.0015^▲^	33.97 ± 19.9	14.11 ± 11.95	2.29	0.0391^▲^
Lower renal artery opening	11.58 ± 7.29	3.69 ± 2.84	3.14	0.0078^▲^	85.3 ± 38.41	64.36 ± 27.63	1.92	0.0768	18.24 ± 12.51	12.18 ± 10.65	0.83	0.4191
Aorta-iliac bifurcation	9.21 ± 5.77	2.3 ± 2.11	3.44	0.0049^▲^	72.2 ± 30.25	52.68 ± 15.62	2.18	0.0497^▲^	11.36 ± 7.12	6.9 ± 7.19	0.87	0.4024
All levels	15.11 ± 10.65	3.45 ± 2.72	7.64	<0.0001	91.1 ± 45.58	54.75 ± 19.33	6.33	<0.0001	27.07 ± 21.82	12.78 ± 11.79	3.5	0.0009
	peak flow (ml/sec)		t	P	net forward volume (ml)		t	P	regurgitant fraction (%)		t	P
	TL	FL			TL	FL			TL	FL		
Celiac trunk	129.39 ± 74.85	63.31 ± 59.34	1.92	0.0761	32.5 ± 18.69	12.9 ± 11.48	2.52	0.0245^▲^	4.46 ± 13.13	22.84 ± 24.47	−2.21	0.0439^▲^
Superior mesenteric artery	103.42 ± 64.72	45.68 ± 79.44	1.45	0.1712	24.58 ± 14.17	10.42 ± 8.84	2.32	0.0371^▲^	7.46 ± 23.76	35.38 ± 30.55	−2.24	0.0433^▲^
Lower renal artery opening	68.6 ± 43.92	47.37 ± 56.81	0.62	0.545	13.16 ± 8.87	8.88 ± 7.39	0.87	0.3985	11.26 ± 17.12	31.53 ± 26.63	−2.37	0.0338^▲^
Aorta-iliac bifurcation	48.88 ± 32.25	25.78 ± 53.89	0.72	0.4875	8.22 ± 5.1	5.02 ± 4.9	0.98	0.3487	14.3 ± 13.83	48.6 ± 34.4	−3.17	0.0081^▲^
All levels	87.57 ± 63.39	46.2 ± 62.95	2.54	0.014	19.61 ± 15.74	9.44 ± 8.85	3.5	0.0009	9.37 ± 17.46	34.13 ± 29.75	−5.01	<0.0001

^▲^Represents that the difference was statistically significant (p < 0.05).

Correlation and regression analysis for size, number of intimal tears, FL thrombosis, and blood flow values

The correlation analysis is provided in Tables 1 and 2 of the online supplementary material. The size of the entries was negatively correlated with the average through-plane velocity, peak velocity magnitude, average net flow, and peak flow in the TL, and positively correlated with the average through-plane velocity, average net flow, and peak flow in the FL. When considering the number of entries, there was a positive correlation with the average through-plane velocity, peak velocity magnitude, average net flow, and peak flow in the TL, and a negative correlation with the peak flow in the FL. If FL displayed thrombosis, the average through-plane velocity and the peak velocity magnitude of the TL increased.

Results of the multiple regression analysis are exhibited in Tables 3 and 4 of the online supplementary material. The results established that the size of the entries had a significant influence on the blood flow values of TL and FL. The size was negatively correlated with the average through-plane velocity, peak velocity magnitude, average net flow, and peak flow of TL, while it was positively correlated with the average through-plane velocity of FL. Further, it was negatively correlated with the peak velocity magnitude of FL.

**Figure 4:**
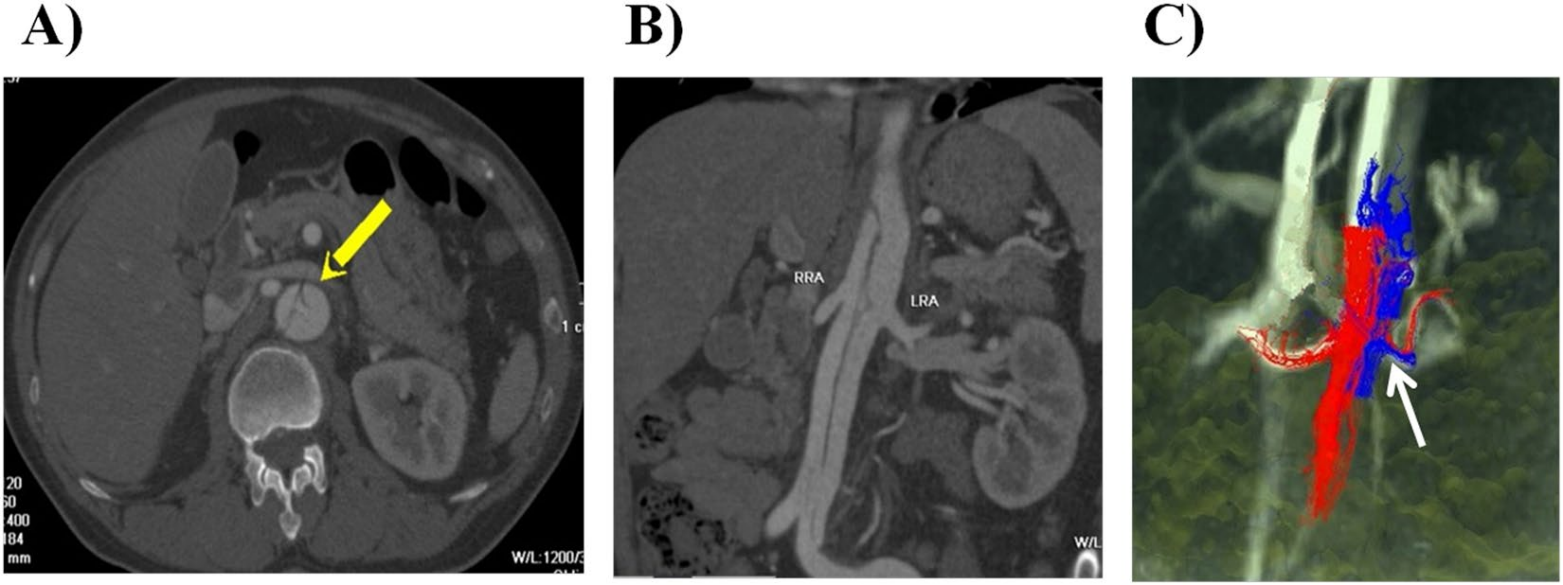
The location and blood flow patterns of the secondary tears in AD. (**A**,**B**) Multiplanar reconstruction images confirming that the right renal artery originated from the TL and the left renal artery from the FL; small secondary tear located at the opening level of the left renal artery. (**C**) Color-coded streamlined visualization of the differences between the TL (red) and FL (blue) in the flow patterns. Due to the presence of the secondary tear, the left renal artery was supplied by the TL and the FL. TL = true lumen, FL = false lumen.

The abdominal aorta, especially the opening level of the renal artery, often demonstrated secondary tears, which proved to be immensely difficult to image. Nonetheless, 4D flow imaging combined with conventional CTA imaging can accurately display the location of the secondary tears and the blood flow patterns in the concerned arteries (Fig. 4).

## Discussion

In this study, we investigated the clinical application of 4D-flow MRI imaging for the qualitative and quantitative analysis of the flow alterations in the TL and FL of abdominal aortic dissections. Our results ascertain that the abdominal aortic blood flow in AD patients varies between the TL and FL, and that the values are directly related to the number and size of intimal tears, as well as to FL thrombosis.

The formation and progression of AD are closely related to the hemodynamic factors and compliance of the aorta. The blood flow velocity, flow rate, pattern, and pressure differences between the TL and FL are the key factors that influence the collapse of the TL and expansion of the FL. 4D-flow imaging can be applied to accurately measure the blood flow velocity, forward and backward flow, blood flow pattern, and regurgitant fraction in AD patients^17,18^. The root cause of the pathological changes related to AD can be discussed with regard to several hemodynamic aspects, and the development of AD can be predicted from the quantitative information on blood flow. Apart from this study, previous reports^11,19^ have also suggested that the hemodynamic changes in the FL are associated with the formation and expansion of the FL.

Traditionally, the most common cardiac magnetic resonance (CMR) flow imaging technique was 2D cine PC CMR with velocity-encoding in a single direction (2D cine PC-CMR)^16,20–22^. However, the usefulness of the technique was limited to the imaging of a single 2D plane. The single velocity-encoding direction is typically selected perpendicular to the 2D plane, which enables calculation of the flow volume through the plane. The invasive thermodilution technique that was widely employed earlier for flow quantification is fraught with inaccuracies due to its underlying assumptions. Unlike Doppler echocardiography or 2D cine PC-CMR, 4D-Flow CMR acquisition includes measurements representing three directions and spatial regions of the flow within the boundaries of the volume imaged. “4D-Flow CMR” refers to phase-contrast CMR incorporating flow-encoding in all three spatial directions that are resolved relative to all three dimensions of space and time along the cardiac cycle (3D + time = 4D)^23^. A major advantage of 4D flow MRI when compared with the 2D version is that the entire abdominal aorta is imaged in a single acquisition of a reasonable length and could be easily integrated with routine clinical MRI studies.

Previous investigations mostly focused on the blood flow in the thoracic aorta^24^. However, this research focused on the blood flow patterns in the distal segments of the thoracic and abdominal aorta. Therefore, it is expected that a comprehensive understanding of the complex hemodynamic changes occurring in patients with AD could be achieved. The aim is to provide quantitative data analysis and visualization of blood flow patterns for guiding the treatment and attaining a superior therapeutic effect. Our work utilized a combination of 4D flow MRI and CTA imaging to analyze the abdominal blood flow in aortic dissection, an enhanced approach that was lacking in earlier studies.

Prior investigations on the disease identified that the persistent presence of the FL was the key factor affecting the progression of AD^4,25^. Toshihisa *et al*.^26^ performed a multi-factor analysis of variance on the factors influencing the growth rate of the FL in chronic AD patients from hemodynamic aspects. The results exposed that the FL blood flow volume was an independent factor affecting the growth rate of FL; the greater the blood flow volume, the more rapid was the growth of FL. It was also suggested that the prognosis depended on the hemodynamic alterations resulting from the pathological changes. According to the Bernoulli energy equation^27^, lesser the velocity of the fluid, greater is its hydrostatic pressure. In our case, the mean FL area was larger than that of the TL at all levels. The mean blood flow velocity in the FL was significantly lower, while the regurgitant fraction was comparatively reduced in the TL. It can hence be assumed that the pressure in the FL is higher than that in the TL. As the TL narrowed and the FL expanded, there was an evident correlation between the TL collapse degree and the FL blood flow velocity. The higher FL pressure, the greater degree of TL collapse, and the increased maximum diameter of the aorta are all risk factors for aortic rupture and organ ischemia^11^. As Tolenaar *et al*.^18^ hypothesized, a region of high pressure in the FL may indicate a probable area of aortic growth arising. The FL carried a greater proportion of the descending aortic flow and was significantly larger than the TL. Moreover, the FL exhibited a relatively higher homogenous pressure gradient along its length, with lower velocities and wall shear stress.

More than two tears are routinely found in patients with AD^28^. These secondary tears are typically located in the vicinity of the visceral arterial branches, especially in proximity to the ostium of the renal artery. The impact of these tears on the dissection flow is yet to be fully understood. Thus, the size and number of intimal entries, and FL thrombus were considered in the correlation and regression analysis for shedding light on this issue. The results revealed that the number and size of the intimal tears had the greatest influence on the hemodynamic parameters. This observation is consistent with the findings from other clinical and experimental studies. Tolenaar *et al*.^18^ reported that the patients with a single entry tear had significantly poorer outcomes than those with multiple tears, while Rudenick *et al*.^29^ stated that small secondary tears caused a larger pressure difference between the TL and FL, with the TL displaying comparatively higher pressures. The researchers further confirmed that the TL received a larger proportion of the flow in the case of smaller tears. In another work, Berguer *et al*.^30^ demonstrated that the pressure difference between the TL and the FL was inversely proportional to the cross-sectional area of the exit tear. Furthermore, the study proved that large primary entry tears lead to low blood flow and flow velocity in the TL. Besides, these parameters were also affected by the increased number of secondary tears and FL thrombosis. In addition, Clough *et al*.^31^ ascertained the potential of 4D PC-MRI as a valuable technique for identifying patients experiencing higher rates of aortic expansion. Multi-modality imaging can effectively analyze the morphological and hemodynamic aspects involved in the prognosis of AD. Further, CT or MRI-derived computational fluid dynamics (CFD) can expose the hemodynamic changes in AD and predict the FL patency or thrombosis^32–34^.

### Limitations

This study does face a few limitations. First, the number of participants was relatively small, and it relied on a single-center experience. Hence, further studies with the inclusion of more cases are needed. Second, the low signal in the FL restricted our analysis of the flow features in the region, and the flow signal could not be differentiated from noise in several instances. Third, additional studies should include a group of patients with medically treated aortic dissection, to identify which flow characteristics distinguish these two groups and hence manage the AD patients in a better way. Furthermore, future work must focus on data acquisition strategies^35^ with an increased velocity-encoding sensitivity spectrum for improved visualization of regions with low flow. Finally, although significant velocity and volume variations between the TL and FL were registered with the use of 4D flow imaging, longitudinal studies with information on patient follow-up are necessary. Such studies are useful in correlating the results of 4D flow imaging with clinical outcomes, specifically with changes in the abdominal blood flow before and after endovascular stent grafting. Therefore, future studies should include long-term follow-up.

## Conclusion

This study has established that 4D flow imaging is a reliable technique for obtaining qualitative and quantitative assessments of the abdominal aortic blood flow in AD, a complex and patient-specific condition. The FL expansion may be attributed to variations in blood flow, pressure, and wall shear stress. Blood flow values were measured to evaluate the disease prognosis. The results demonstrate the potential of 4D flow imaging in identifying patients with higher rates of aortic expansion. The findings would allow us to determine the predictive value of these measurements and assess the future role of 4D flow MRI in risk stratification of AD patients and in deciding appropriate treatment methods. The imaging technique is especially useful as it is a non-invasive technique for determining blood flow changes in patients with AD.

## Electronic supplementary material

Supplementary Tables
