# Supplementary material for: Quantitative Study of Abdominal Blood Flow Patterns in Patients with Aortic Dissection by 4-Dimensional Flow MRI
Source: Sci Rep. 2018 Jun 14;8:9111. doi: 10.1038/s41598-018-27249-9 (PMC6002546; doi:10.1038/s41598-018-27249-9)
Supplement: Supplementary file 1 — Supplementary Tables [file 41598_2018_27249_MOESM1_ESM.docx]

**Supplementary Table 1.** **The correlation between size, number of intimal tears, false lumen thrombosis and** **blood flow values in TL**

| Variables | average th-plane velocity | | peak velocity magnitude | | average net flow | | peak flow | |
| --- | --- | --- | --- | --- | --- | --- | --- | --- |
|  | r | P | r | P | r | P | r | P |
| Size of entries (mm) | -0.46 | 0.0001 | -0.61 | <0.0001 | -0.35 | 0.0042 | -0.46 | 0.0002 |
| Number of entries | 0.30 | 0.0145 | 0.44 | 0.0003 | 0.33 | 0.0080 | 0.34 | 0.0059 |
| False lumen thrombus | 0.36 | 0.0032 | 0.34 | 0.0055 | 0.14 | 0.2809 | 0.06 | 0.6262 |

r represents pearson correlation coefficient. P<0.05 indicated that the difference was statistically significant.

**Supplementary Table 2.** **The correlation between size, number of intimal tears, false lumen thrombosis and blood flow values in FL**

| Variables | average th-plane velocity | | peak velocity magnitude | | average net flow | | peak flow | |
| --- | --- | --- | --- | --- | --- | --- | --- | --- |
|  | r | P | r | P | r | P | r | P |
| Size of entries (mm) | 0.36 | 0.0066 | -0.26 | 0.0542 | 0.38 | 0.0041 | 0.38 | 0.0035 |
| Number of entries | -0.14 | 0.3166 | 0.06 | 0.6816 | -0.12 | 0.3969 | -0.30 | 0.0225 |
| False lumen thrombus | -0.23 | 0.0841 | -0.05 | 0.6979 | -0.25 | 0.0642 | -0.25 | 0.0642 |

r represents pearson correlation coefficient. P<0.05 indicated that the difference was statistically significant.

**Supplementary Table 3. The results of regression analysis between size, number of intimal tears, false lumen thrombosis and blood flow values in TL**

| Variables | average th-plane velocity | | peak velocity magnitude | | average net flow | | peak flow | |
| --- | --- | --- | --- | --- | --- | --- | --- | --- |
|  | β | P | β | P | β | P | β | P |
| Size of entries (mm) | -0.68 | 0.0221^▲^ | -3.98 | 0.0006^▲^ | -1.46 | 0.0262^▲^ | -6.03 | 0.001^▲^ |
| Number of entries | 1.12 | 0.5111 | 11.56 | 0.081 | 2.49 | 0.5116 | 7.76 | 0.4525 |
| False lumen thrombus | 5.62 | 0.0541 | 17.67 | 0.1116 | -2.93 | 0.6454 | -22.80 | 0.1911 |

**Supplementary Table 4. The results of regression analysis between size, number of intimal tears, false lumen thrombosis and blood flow values in FL**

| Variables | average th-plane velocity | | peak velocity magnitude | | average net flow | | peak flow | |
| --- | --- | --- | --- | --- | --- | --- | --- | --- |
|  | β | P | β | P | β | P | β | P |
| Size of entries (mm) | 0.17 | 0.0451^▲^ | -1.35 | 0.0264^▲^ | 0.62 | 0.0817 | 3.17 | 0.0887 |
| Number of entries | -0.13 | 0.7874 | -1.16 | 0.749 | -1.34 | 0.5333 | -19.04 | 0.0928 |
| False lumen thrombus | -0.10 | 0.9011 | -9.06 | 0.1372 | -3.18 | 0.3756 | -3.42 | 0.8547 |

β represents regression coefficient. ^▲^indicated that the independent variable has a statistically significant effect on the relevant blood flow parameters. P<0.05
